# Comparison of arthroscopic and open Brostrom-Gould surgery for chronic ankle instability: a systematic review and meta-analysis

**DOI:** 10.1186/s13018-023-04292-5

**Published:** 2023-11-14

**Authors:** Binzhi Zhao, Qingnan Sun, Xiaopei Xu, Yang Liu, Yanrui Zhao, Yulin Gao, Junlin Zhou

**Affiliations:** Department of Orthopedic Surgery, Beijing Chaoyang Hospital, Capital Medical University, 8 Gongren Tiyuchang Nanlu, Chaoyang District, Beijing, 100020 People’s Republic of China

**Keywords:** Chronic lateral ankle instability, Open, Arthroscopic, Brostrom-Gould, Repair, Meta-analysis

## Abstract

**Background:**

Approximately 20% of acute ankle sprains progress to chronic lateral ankle instability (CLAI) requiring surgical intervention. There has been growing interest among surgeons regarding whether arthroscopic techniques can replace open Brostrom-Gould surgery in treating CLAI. The purpose of this study was to pool the results of multiple studies comparing the treatment effects of these two fixation approaches.

**Methods:**

Our study involved thorough searches across multiple electronic databases, including PubMed, Cochrane, Embase, and Web of Science, to identify all relevant publications on CLAI that were repaired using the arthroscopic or open Broström-Gould technique. Through a comprehensive meta-analysis, we evaluated several outcomes, including post-operative function, radiological measurements, complications, and time efficiency.

**Result:**

A total of 686 patients from 11 studies were included in the analysis. Among them, 351 patients underwent open repair, and 335 underwent arthroscopic Brostrom-Gould surgery. The present study revealed that arthroscopic and open Brostrom-Gould techniques demonstrated no significant differences in talar tilt, talar anterior translation, complication rate, and time to return to previous level of activity. Furthermore, no significant differences were observed in AOFAS, K–P, VAS, and Tegner scores at the 2-year follow-up. However, significant differences were noted between the two surgical approaches in terms of early weight-bearing (WMD = − 1.33 weeks, 95% CI = [− 1.91, − 0.76], *P* = 0.17, I^2^ = 40%), as well as AOFAS scores (WMD = 1.00, 95% CI = [0.05, 1.95], *P* = 0.73, I^2^ = 0%), K–P scores (WMD = 1.57, 95% CI = [0.49, 2.64], *P* = 0.15, I^2^ = 47%), and VAS scores (WMD = − 0.15, 95% CI = [− 0.60, 0.29], *P* < 0.08, I^2^ = 61%) within the first postoperative year.

**Conclusions:**

Our findings support that arthroscopic repair yields comparable outcomes to open surgery. Consequently, we advocate for adopting arthroscopic repair as a preferred alternative to the conventional open Broström-Gould procedure for treating chronic lateral ankle instability.

## Introduction

The most common injury to the ankle joint is an ankle sprain, comprising for approximately 85% of all ankle injuries, with the majority being lateral inversion sprains [1]. The injury primarily involves the anterior talofibular ligament (ATFL), and the extent of damage to the ATFL ranges from mild stretching to severe complete tear [2]. Most patients achieve favorable treatment outcomes with temporary activity restriction and functional rehabilitation. However, a study indicated that around 20% of individuals who suffer from acute ankle sprains may develop chronic lateral ankle instability (CLAI), necessitating potential surgical treatment [3].

Some studies have demonstrated that the Broström-Gould technique, which involves repairing the ATFL repair with an inferior extensor retinaculum augmentation, has shown favorable outcomes in restoring lateral ankle stability [4–6]. The open Broström-Gould technique has long been considered the gold standard for treating CLAI [7–9]. Recently, there has been a rising interest in adopting arthroscopic Brostrom-Gould as a substitute for conventional open Brostrom-Gould surgery. Compared to open surgery, the arthroscopic technique has the advantage of easily detecting intra-articular abnormalities and being able to address them while repairing the ligament [10–12]. It has been reported to achieve similar or even superior clinical scores and faster rates of motion recovery [13, 14]. Additionally, it may facilitate an accelerated postoperative rehabilitation process [15]. However, a study suggests that while arthroscopic surgery is popular, there is no evidence proving it to be more beneficial than traditional open surgery [16].

There is ongoing controversy regarding the potential of arthroscopic techniques to replace open Brostrom-Gould surgery as a treatment option for CLAI. Therefore, this meta-analysis aims to pool the results of multiple studies comparing the treatment effects of these two fixation approaches.

## Materials and methods

### Search strategy

The present study followed the PRISMA (Preferred Reporting Items for Systematic Reviews and Meta-Analyses) guidelines for reporting systematic reviews and meta-analyses [17]. We registered the review protocol in the PROSPERO database (CRD42023406427) [18]. The last search date was July 5, 2023, and the PubMed, the Cochrane Library, Embase and Web of Science databases were searched. The search procedure is based on the following keywords: (“Arthroscopic” OR “Arthrosc*” OR “Minimally invasive surg*” OR “Endoscop*”) AND (“Brostrom” OR “Modified Brostrom” OR “Brostrom-Gould” OR “inferior extensor retinaculum” OR “IER”). We initially screened the title and abstract of each article to determine eligibility, followed by a full-text assessment of those meeting the criteria. Moreover, we thoroughly examined the references cited in the included articles for completeness.

### Inclusion and exclusion criteria

This systematic review and meta-analysis included comparative studies on arthroscopic and open Bröstrom-Gould procedure in recurrent ankle sprains and chronic ankle instability, defined as persisting instability symptoms after 6 months of conservative treatment, positive anterior drawer test, and isolated grade III chronic ATFL and CFL injuries confirmed by magnetic resonance imaging (MRI). Studies including patients with previous ankle fractures, affected ankle surgery, severe ankle arthritis, combined neuromuscular diseases, and generalized ankle laxity were excluded. Arthroscopic Bröstrom-Gould procedure was considered the experimental group, while open repair was the control intervention. Outcomes measured included functional scores (AOFAS, K–P VAS, and Tegner scores), radiological outcome (anterior drawer and talar tilt), complication rate, duration of operation, time to return to weightbearing, and sport.

Additionally, we included studies with high quality and excluded cases, reviews, and studies with low homogeneity. There were no restrictions on language.

### Quality assessment

Two authors evaluated the quality of the included studies using the Cochrane Reviewer’s Handbook [19]. This study assessed the risk of bias in seven domains, which include random sequence generation, allocation concealment, blinding of participants and personnel, blinding of outcome assessment, incomplete outcome data, selective reporting, and other sources of bias. Each criterion was assessed for low, unclear, or high degree of bias. Non-randomized controlled trials are evaluated according to Newcastle–Ottawa Scale (NOS), which mainly includes selection, comparison and outcome [20]. Research scoring seven or more points is considered to be of high quality. The assessments of the included studies were conducted independently by two reviewers (B.Z.Z. and Q.N.S.), and any disagreements were resolved by consulting a third reviewer (J.L.Z.).

### Data extraction

Relevant information from the included literature, including first author, publication date, sample size, patient age and gender, surgical approach, and follow-up time, was extracted independently by two reviewers. The outcome measures were the AOFAS scores, K–P scorers, VAS pain scores, Tegner scores, anterior drawer, talar tilt, complication rate, duration of operation, time to return to weightbearing and sport. When standard deviations were missing from studies, we attempted to contact the authors of articles by email to obtain relevant metrics. Medians and ranges were converted without means and SDs, as Wan et al. recommended [21]. Disagreements during the extraction process were resolved through consultation with a third investigator (J.L.Z).

### Statistical analysis

Data were analyzed using Review Manager (RevMan 5.4.1, Nordic Cochrane Center, Copenhagen, Denmark) for this meta-analysis. Mean difference (MD) with 95% confidence intervals (95% CI) was used for continuous data analysis, while odds ratios (OR) with 95% CI were used for dichotomous data analysis. To determine heterogeneity, we used the I^2^ tests. If I^2^ > 50% and *P* < 0.10, it indicates high heterogeneity. In cases of significant heterogeneity, we applied random-effect models. Conversely, we used fixed-effect models when heterogeneity was low.

## Results

### Study selection

**Fig. 1:**
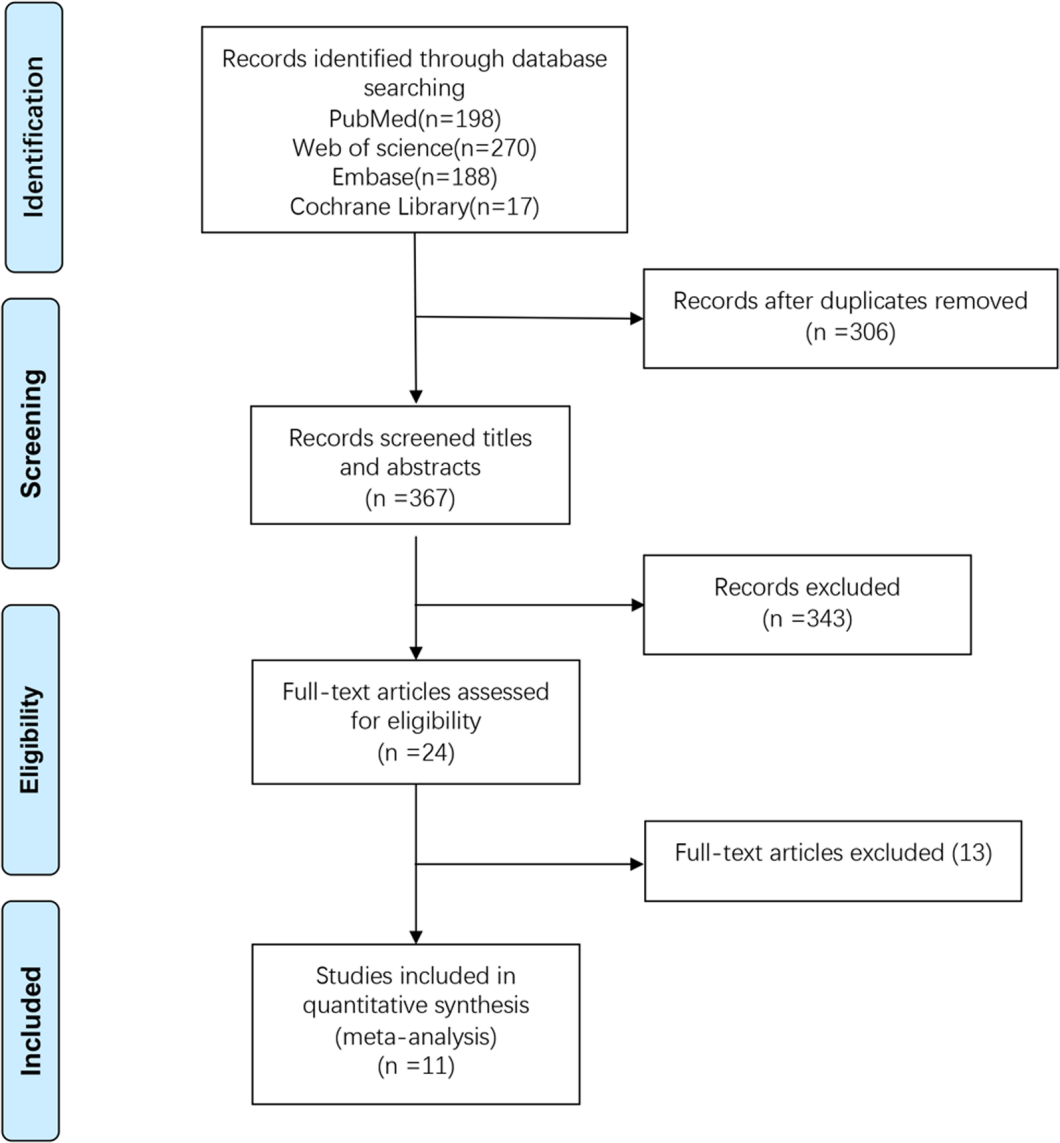
Flow diagram of screening the included studies in the meta-analysis

The PRISMA flow diagram (Fig. 1) illustrates the literature search and selection process. We identified 673 relevant articles by searching electronic databases. Eleven articles were selected for this meta-analysis, all of which compared the effect of arthroscopic and open Brostrom-Gould procedures for the treatment of CLAI.

### Study characteristics

The meta-analysis comprised eleven studies that compared the efficacy of arthroscopic and open Brostrom-Gould procedures for CLAI. These studies included two randomized controlled trials, two prospective and seven retrospective cohort studies, with a total of 686 patients (395 males and 291 females), of which 335 underwent arthroscopic repair, and 351 underwent open Brostrom-Gould (Table 1).

**Table 1:** General characteristics of the included studies

References	Study design	Surgical approach	Number	Male/female	Age	BMI (Kg/m^2^)	Follow-up (months)
Baek [22]	RCC	Open	34	23/11	21.1 ± 3.5	24.1 ± 3.5	70.9 ± 39.0
		Arthroscopic	31	20/11	22.9 ± 4.4	25.2 ± 3.6	28.7 ± 5.4
Wang [23]	PCS	Open	31	10/21	28.6 ± 8.1	22.9 ± 5.1	44.9 ± 48.6
		Arthroscopic	30	12/18	27.2 ± 7.7	23.8 ± 4.7	57.7 ± 53.7
Wang [24]	RCC	Open	50	34/16	31.92 ± 4.77	22.38 ± 2.29	23.52 ± 8.37
		Arthroscopic	49	32/17	31.71 ± 4.99	21.84 ± 2.4	25.0 ± 8.48
Hou [25]	RCT	Open	34	17/17	28.6 ± 4.8	21.7 ± 2.5	22.5 ± 19.6
		Arthroscopic	36	17/19	28.3 ± 5.4	21.0 ± 3.1	19.8 ± 17.9
Zhou [13]	RCC	Open	36	23/13	31.36 ± 7.79	23.63 ± 2.64	33.06 ± 6.82
		Arthroscopic	31	20/11	33.42 ± 6.4	24.42 ± 1.87	29.69 ± 3.40
Woo [26]	RCC	Open	26	16/10	31.5 ± 10.3	27.2 ± 5.2	12
		Arthroscopic	26	16/10	33.4 ± 10.6	26.6 ± 4.5	12
Yi [27]	RCC	Open	30	22/8	37.3	NA	26
		Arthroscopic	35	24/11	39.3	NA	26
Rigby [1]	RCC	Open	32	14/18	37.73 (9–72)	NA	44.4
		Arthroscopic	30	9/21	47.89 (14–83)	NA	15.6
Li [28]	PCS	Open	37	29/8	28.7 ± 8.7	23.9 ± 2.5	35.5 ± 9.9
		Arthroscopic	23	18/5	30.3 ± 10.1	23.3 ± 2.9	39.7 ± 10.3
Yeo [29]	RCT	Open	23	12/11	34.3 (17–52)	NA	12
		Arthroscopic	25	7/18	35.2 (19–54)	NA	12
Matsui [30]	RCC	Open	18	8/10	24 (13–56)	NA	12
		Arthroscopic	19	12/7	28 (8–59)	NA	12

*RCC* Retrospective case–cohort; *PCS* Prospective case-cohort; *RCT* Randomized controlled trial

### Risk-of-bias assessment

**Fig. 2:**
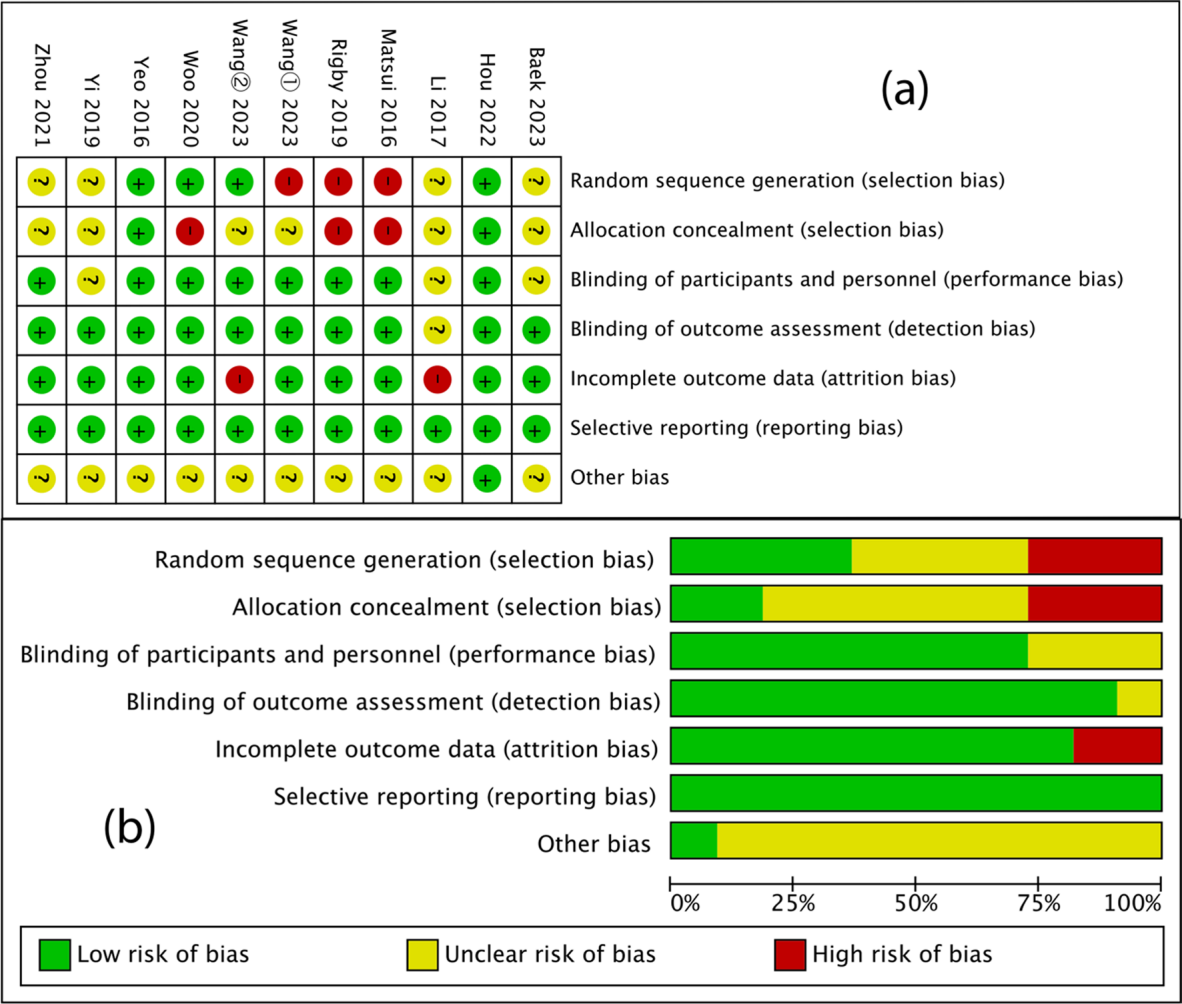
Review authors’ judgments about each risk of bias item for each included study. **a**. Risk of bias summary; **b**. risk of bias graph presented as percentages

The risk of bias items for each included study is shown in Fig. 2. Furthermore, the seven retrospective and two prospective cohort studies were evaluated using the Newcastle–Ottawa Scale and were all rated as high quality, with scores ranging from 7 to 9, as shown in Table 2.

**Table 2:** Quality assessment for the nine cohort studies according to Newcastle–Ottawa scale (NOS)

Study	Year	S				C		E			Total score
		S1	S2	S3	S4	C1	C2	E1	E2	E3	
Baek [22]	2023	★	★	★	★	★	★	★	★	★	9
Wang [23]	2023	★	★	★	★	★	★	★	★	★	9
Wang [24]	2023	★	★	★	★	★	★	★	★	–	8
Zhou [13]	2021	★	★	★	★	★	★	★	★	★	9
Woo [26]	2020	★	★	★	★	★	★	★	★	★	9
Yi [27]	2019	★	★	★	★	★	–	★	★	★	8
Rigby [1]	2019	★	★	★	★	★	–	★	★	★	8
Li [28]	2017	★	★	★	★	★	–	★	★	★	8
Matsui [30]	2016	★	★	★	★	★	–	★	★	★	8

★: The scale uses a star symbol (★) to represent points, and each star represents one point

S selection, C comparability, E exposure. S1 representativeness of the exposed cohort, S2 selection of the nonexposed cohort, S3 ascertainment of exposure, S4 demonstration that outcome of interest was not present at start of study. C1 comparability of controls for the most important factor, C2 comparability of controls for a second important factor. E1 assessment of outcome, E2 was follow-up long enough for outcomes to occur, E3 adequacy of follow-up of cohorts

### Functional outcome

#### AOFAS score

**Fig. 3:**
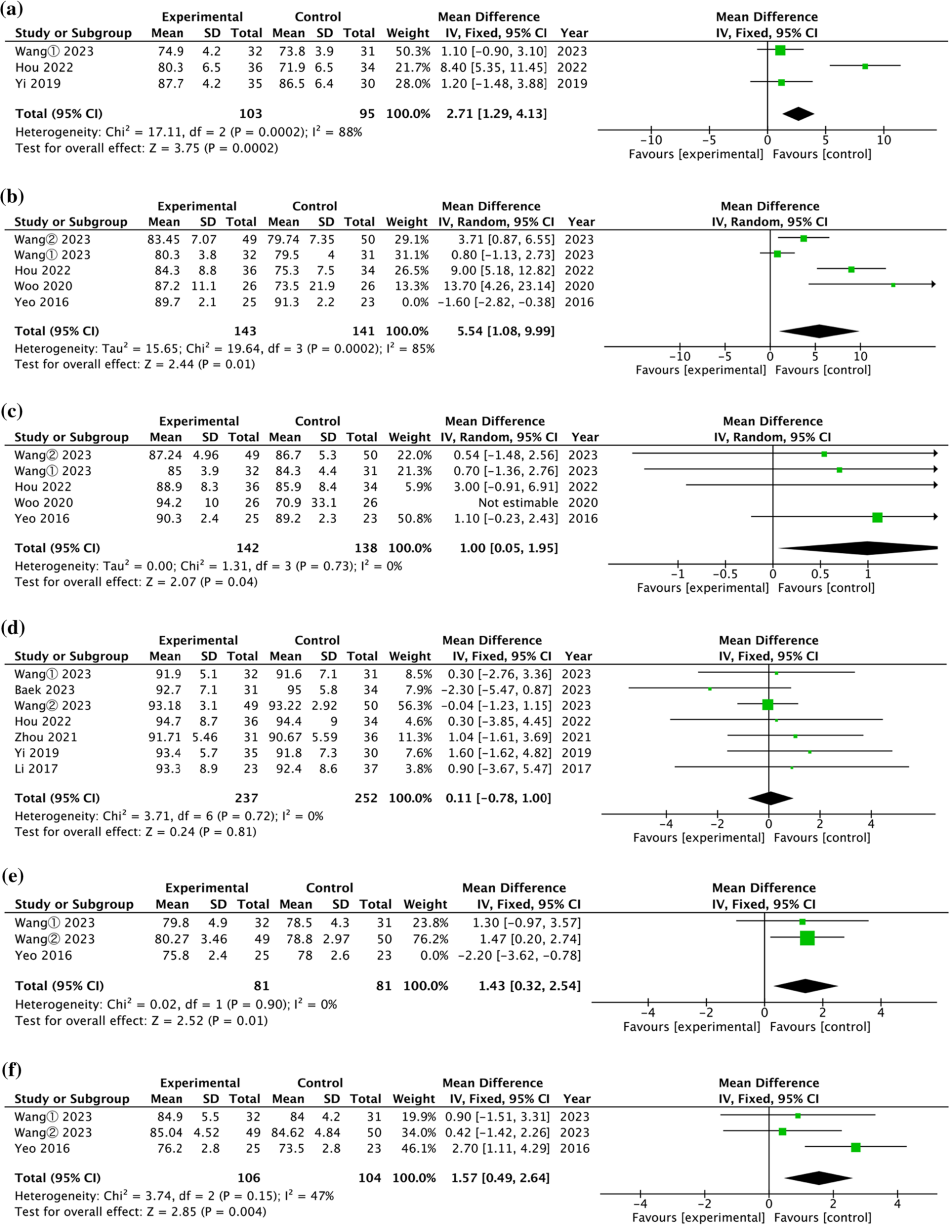

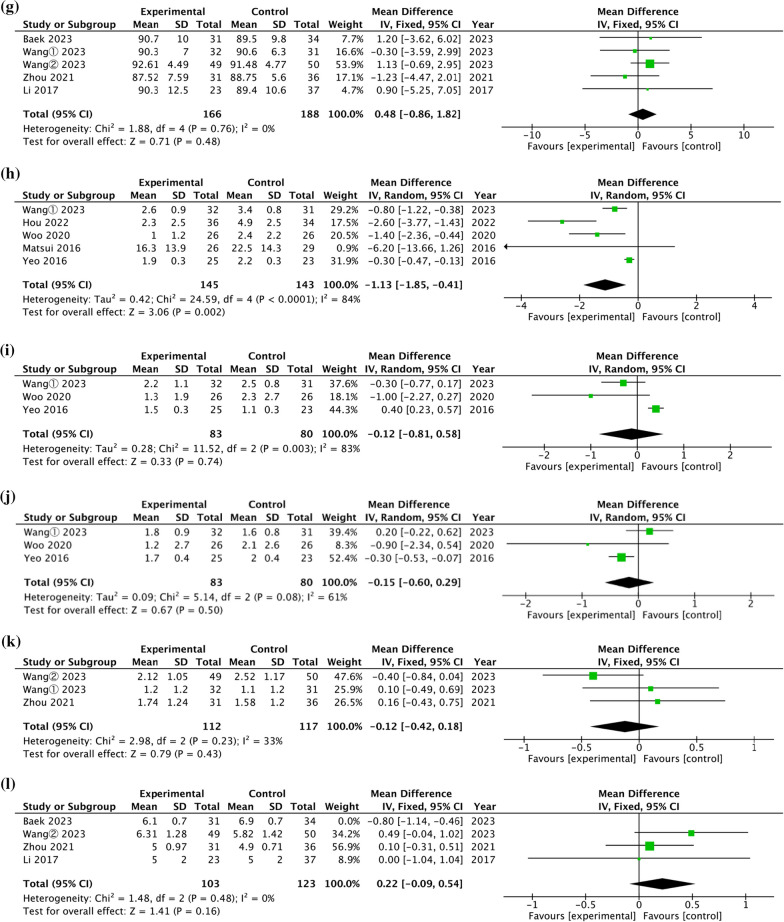
Forest plot of comparison of functional outcome. **a** 3 months postoperative AOFAS score. **b** 6 months postoperative AOFAS score. **c** 12 months postoperative AOFAS score. **d** 2 years postoperative AOFAS score. **e** 6 months postoperative K–P score. **f** 12 months postoperative K–P score. **g** 2 years postoperative K–P score. **h** Perioperative VAS score. **i** 6 months postoperative VAS score. **j** 12 months postoperative VAS score. **k** 24 months postoperative VAS score. **l** Tegner scores final follow-up

Three studies evaluated the postoperative 3-month AOFAS score of CLAI treated with open and arthroscopic Brostrom-Gould surgery [23, 25, 27]. The pooled results in Fig. 3a indicated no significant difference between two surgical techniques (WMD = 3.46, 95% CI = [− 0.85, 7.77], *P* = 0.12), with high heterogeneity (*P* = 0.0002, I^2^ = 88%). Four studies evaluated the postoperative 6-month AOFAS score of CLAI treated with open and arthroscopic Brostrom-Gould surgery [23–25, 31]. The pooled results in Fig. 3b demonstrated a significant difference between two surgical techniques (WMD = 5.54, 95% CI = [1.08, 9.99], *P* = 0.11), with high heterogeneity (*P* = 0.02, I^2^ = 67%). Four studies evaluated the postoperative 1-year AOFAS score of CLAI treated with open and arthroscopic Brostrom-Gould surgery [23–25, 29]. The pooled results in Fig. 3c demonstrated a significant difference between two surgical techniques (WMD = 1.00, 95% CI = [0.05, 1.95], *P* = 0.04), with no heterogeneity (*P* = 0.73, I^2^ = 0%).

Seven studies evaluated the postoperative 2-year AOFAS score of CLAI treated with open and arthroscopic Brostrom-Gould surgery [13, 23–25, 27, 28, 32]. The pooled results in Fig. 3d indicated no significant difference between two surgical techniques (WMD = 0.11, 95% CI = [− 0.78, 1.00], *P* = 0.81), with no heterogeneity (*P* = 0.72, I^2^ = 0%).

#### K–P score

Two studies evaluated the postoperative 6-month K–P score of CLAI treated with open and arthroscopic Brostrom-Gould surgery [23, 24, 29]. The pooled results in Fig. 3e demonstrated a significant difference between two surgical techniques (WMD = 1.43, 95% CI = [0.32, 2.54], *P* = 0.01), with no heterogeneity (*P* = 0.90, I^2^ = 0%).

Three studies evaluated the postoperative 1-year K–P score of CLAI treated with open and arthroscopic Brostrom-Gould surgery [23, 29, 31]. The pooled results in Fig. 3f demonstrated a significant difference between two surgical techniques (WMD = 1.57, 95% CI = [0.49, 2.64], *P* = 0.004), with low heterogeneity (*P* = 0.15, I^2^ = 47%).

Five studies evaluated the postoperative 2-year K–P score of CLAI treated with open and arthroscopic Brostrom-Gould surgery [13, 23, 24, 28, 32]. The pooled results in Fig. 3g demonstrated no significant difference between two surgical techniques (WMD = 0.48, 95% CI = [− 0.86, 1.82], *P* = 0.48), with no heterogeneity (*P* = 0.76, I^2^ = 0%).

#### VAS pain score

Five studies evaluated the perioperative VAS score of CLAI treated with open and arthroscopic Brostrom-Gould surgery [23, 25, 29–31]. The pooled results in Fig. 3h demonstrated a significant difference between two surgical techniques (WMD = − 1.13, 95% CI = [− 1.85, − 0.41], *P* = 0.002), with high heterogeneity (*P* < 0.0001, I^2^ = 84%). Three studies evaluated the 6-month VAS score of CLAI treated with open and arthroscopic Brostrom-Gould surgery [23, 29, 31]. The pooled results in Fig. 3i demonstrated no significant difference between two surgical techniques (WMD = − 0.12, 95% CI = [− 0.81, 0.58], *P* = 0.74), with high heterogeneity (*P* < 0.003, I^2^ = 83%). Three studies evaluated the 1-year VAS score of CLAI treated with open and arthroscopic Brostrom-Gould surgery [23, 29, 31]. The pooled results in Fig. 3j demonstrated no significant difference between two surgical techniques (WMD = − 0.15, 95% CI = [− 0.60, 0.29], *P* = 0.50), with high heterogeneity (*P* < 0.08, I^2^ = 61%).

Three studies evaluated the 2-year VAS score of CLAI treated with open and arthroscopic Brostrom-Gould surgery [13, 23, 24]. The pooled results in Fig. 3k demonstrated no significant difference between two surgical techniques (WMD = − 0.12, 95% CI = [− 0.42, 0.18], *P* = 0.43), with low heterogeneity (*P* = 0.23, I^2^ = 33%).

#### Tegner

Three studies evaluated the Tegner scores at final follow-up [13, 24, 28]. The pooled results in Fig. 3L demonstrated no significant difference in the postoperative 2-year VAS score between two surgical techniques (WMD = 0.22, 95% CI = [− 0.09, 0.54], *P* = 0.16), with no heterogeneity (*P* = 0.48, I^2^ = 0%).

### Radiological outcome

**Fig. 4:**
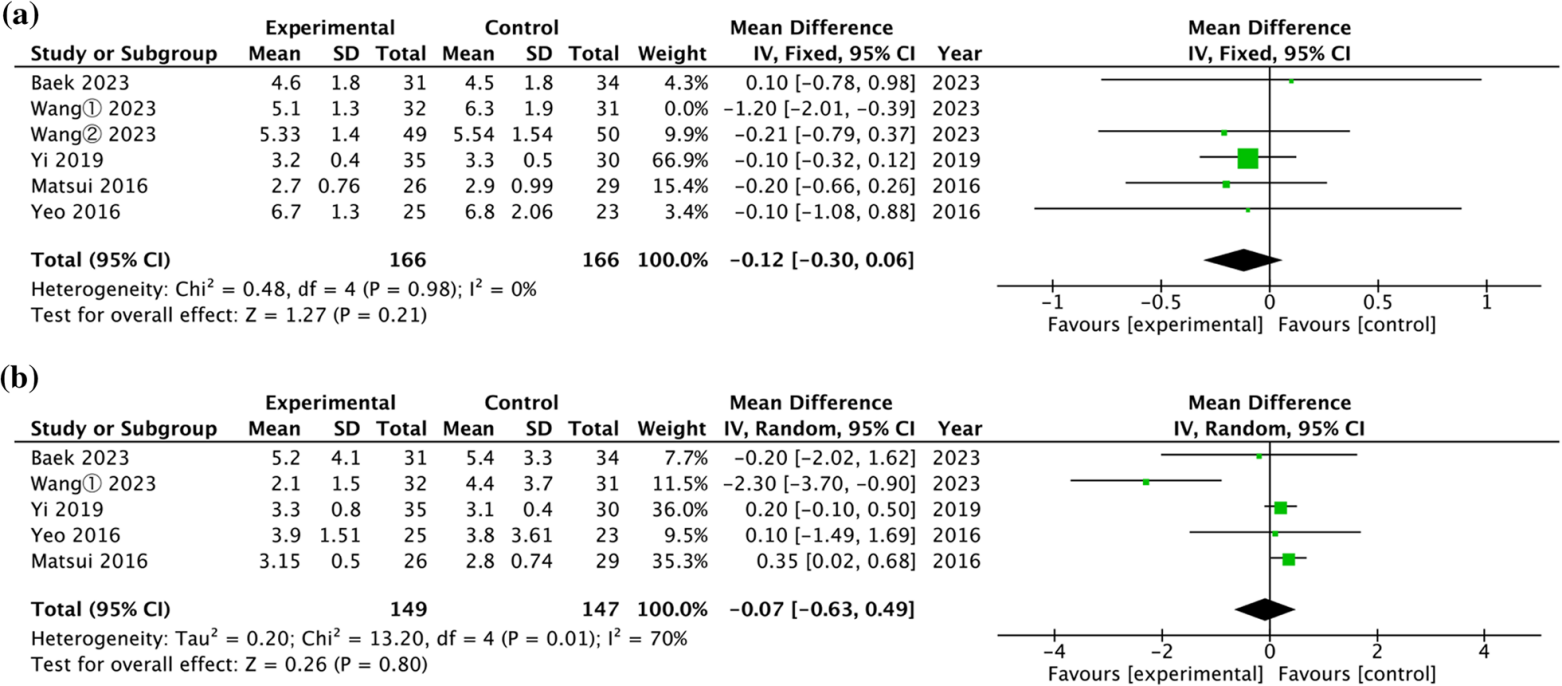
Forest plot of comparison of radiological outcome. **a** Talar anterior translation. **b** Talar tilt

*Anterior talar translation* Five studies compared the postoperative anterior talar translation [23, 24, 27, 29, 30, 32]. A pooled analysis of the data in Fig. 4a found no significant difference between the two fixation approaches (WMD = − 0.12 mm, 95% CI = [− 0.30, 0.06], *P* = 0.21), with no heterogeneity (*P* = 0.98, I^2^ = 0%).

### Talar tilt

Five studies compared the postoperative talar tilt [23, 27, 29, 30, 32]. A pooled analysis of the data in Fig. 4b found no significant difference between the two fixation approaches (WMD = − 0.07 mm, 95% CI = [− 0.63, 0.49], *P* = 0.80), with high heterogeneity (*P* = 0.01, I^2^ = 70%).

### Time efficiency

**Fig. 5:**
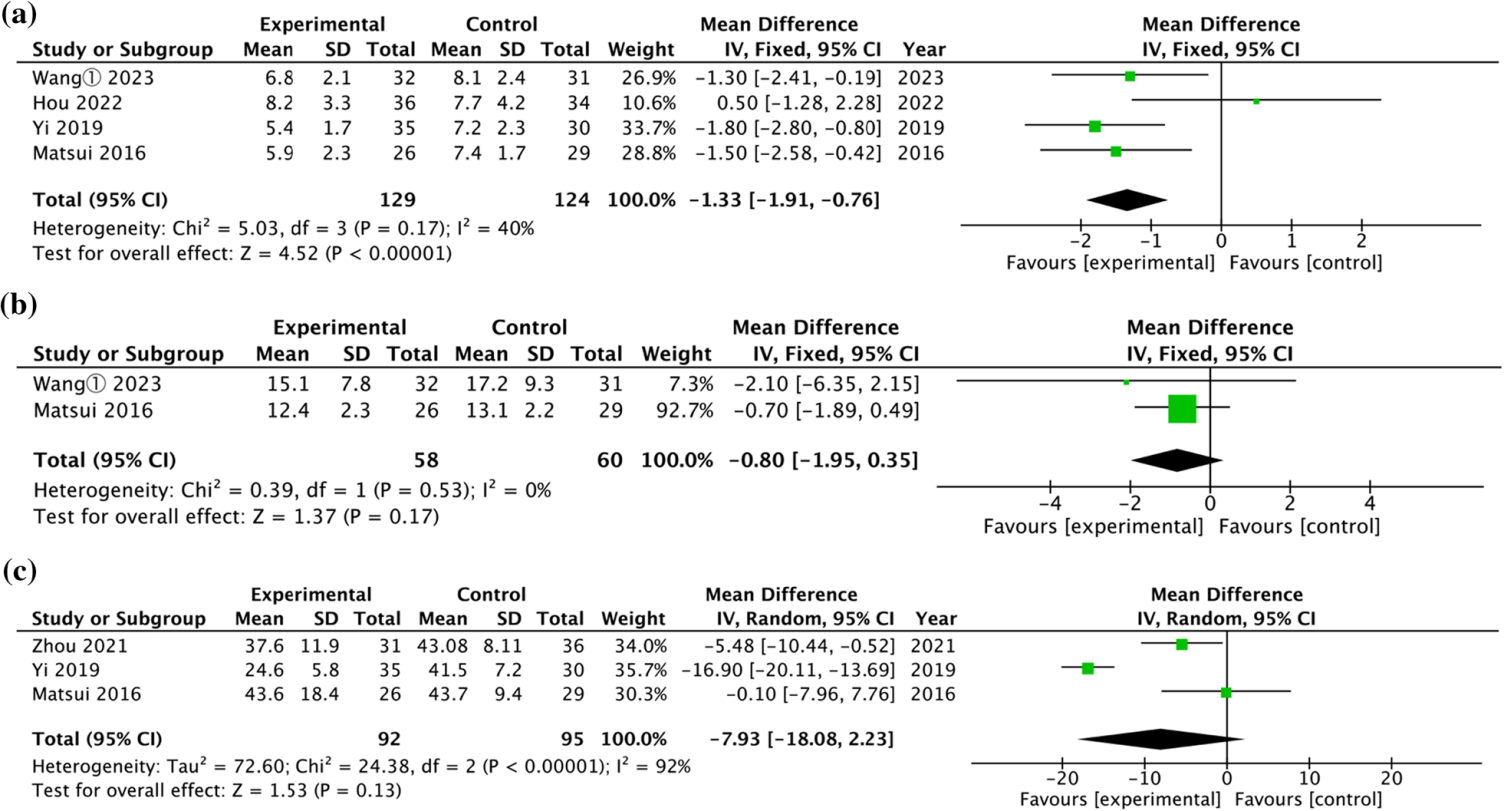
Forest plot of comparison of time efficiency. **a** Time to return to weightbearing**. b** Time to return to sports. **c** Operative time

Four studies compared the time to return to weightbearing [23, 25, 27, 30]. A pooled analysis of the data in Fig. 5a found a significant difference between two surgical techniques (WMD = − 1.33 weeks, 95% CI = [− 1.91, − 0.76], *P* < 0.00001), with low heterogeneity (*P* = 0.17, I^2^ = 40%).

Two studies evaluated the time to return to sports of CLAI treated with open and arthroscopic Brostrom-Gould surgery [23, 30]. The pooled results in Fig. 5b demonstrated no significant difference between two surgical techniques (WMD = − 0.80 weeks, 95% CI = [− 1.95, 0.35], *P* = 0.17), with no heterogeneity (*P* = 0.53, I^2^ = 0%).

Three studies evaluated the operative time of CLAI treated with open and arthroscopic Brostrom-Gould surgery [13, 27, 30]. The pooled results in Fig. 5c demonstrated no significant difference between two surgical techniques (WMD = − 7.93 min, 95% CI = [− 18.08, 2.23], *P* = 0.13), with high heterogeneity (*P* < 0.00001, I^2^ = 92%).

### Complications

**Fig. 6:**
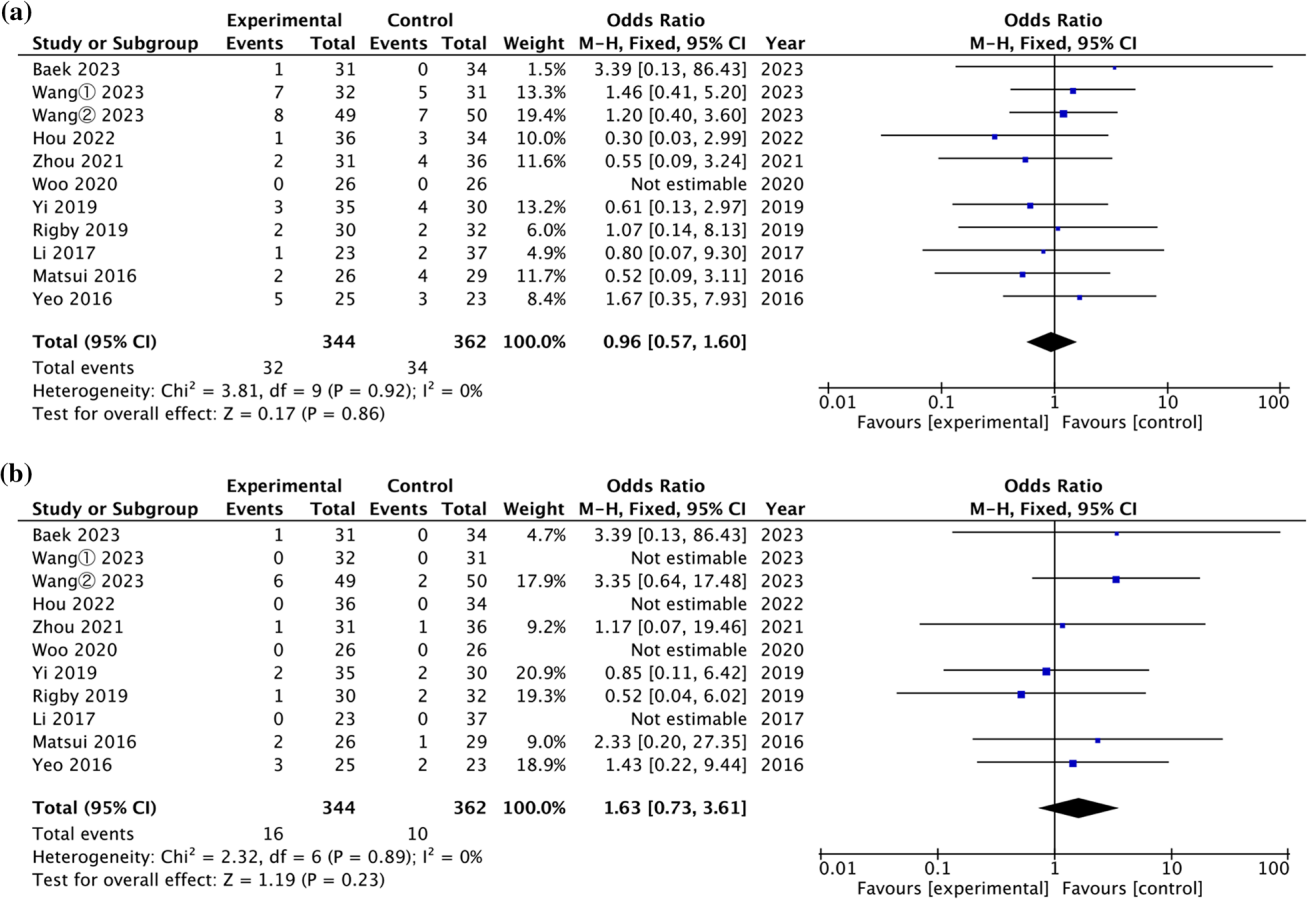
Forest plot of comparison of complications. **a** Total complication **b** Nerve complication

*Total complications* All eleven studies reported total complications [1, 13, 23–25, 27–32]. The pooled analysis in Fig. 6a found no significant difference in total complications between the two fixation approaches (OR = 0.96, 95% CI = [0.57, 1.60], *P* = 0.86), with no heterogeneity (*P* = 0.92, I^2^ = 0%).

*Nerve complications* All eleven studies reported nerve complications [1, 13, 23–25, 27–32]. The pooled analysis in Fig. 6b found no significant difference in nerve complications between the two fixation approaches (OR = 1.63, 95% CI = [0.73, 3.61], *P* = 0.23), with no heterogeneity (*P* = 0.89, I^2^ = 0%).

## Discussion

The results of our study demonstrated comparable outcomes between the two fixation approaches in terms of talar tilt, anterior talar translation, final follow-up AOFAS, K–P, VAS, and Tegner scores, complication rate, time to return to sports, and operation time. However, significant differences were observed in AOFAS, K–P, and VAS scores within 12 months postoperatively. Additionally, there were significant differences between the two surgical approaches regarding early weight-bearing. Arthroscopic repair allowed early postoperative activity.

Two previous studies have compared arthroscopic and open Broström-Gould techniques [10, 33]. However, the earlier study included only four studies, and the other included eight studies with low homogeneity and significant heterogeneity in their results. With an increasing number of recent comparative studies on these two techniques, we included more relevant studies. This improves the reliability of our findings.

### Radiological outcome

Our results demonstrate no significant difference between the two surgical approaches in postoperative talar tilt and anterior talar translation. Arthroscopic modified Brostrom operation demonstrated favorable outcomes comparable to open repair, as assessed by biomechanical activity and clinical measures [34–36]. In a study conducted by Kim et al. involving 28 ankles, the final follow-up AOFAS score showed a significant increase compared to the preoperative score, and there was a notable improvement in the anterior drawer test score [37]. In a study conducted by Nery et al. [2] involving 38 patients, it was found that the average AOFAS score at the final follow-up assessment was 90, and there were no significant differences in radiographic findings between the injured side and the contralateral side. In a biomechanical study by Lee et al. [36], Eleven matched pairs of human cadaver specimens were utilized to compare the outcomes of arthroscopic Broström-Gould operation using a suture anchor and open modified Brostrom surgery. The study evaluated parameters such as degrees to failure, torque to failure, and stiffness. The results showed that the two surgical approaches had similar parameters with no significant differences. Giza et al. [35] also reported similar findings, which are consistent with Lee et al. These two studies demonstrated that the arthroscopic and open approaches to Broström-Gould operation resulted in comparable postoperative strength and stiffness.

### Functional outcomes scores

The previous meta-analysis compared the changes in functional scores over time, including AOFAS, K–P, and VAS scores. However, the analysis was restricted to a one-year follow-up period [33]. In our study, we compared the changes over two years. The results indicated that the arthroscopic group outperformed the open group regarding AOFAS, K–P, and VAS scores within one year of follow-up. However, after a two-year follow-up, our results showed no significant difference in scores between the two fixation approaches.

The K–P score was suggested in 1991 to assess ankle function, explicitly focusing on ankle instability. Despite its widespread use, the validity of the K–P score has not been subjected to the same rigorous scrutiny as the AOFAS score [38]. As one of the most commonly used functional scores in clinical practice, the AOFAS score still requires additional validation studies to determine the reliability and clinical significance, especially in the case of minimal clinically significant differences. In their study, Nery et al. [2] examined 28 patients who received arthroscopic Brostrom-Gould repair for CLAI. The mean follow-up period was 9.8 years, and the average AOFAS score at the last follow-up was 90. Almost all patients (94.7%) achieved excellent and good postoperative AOFAS scores. Acevedo and Mangone’s study encompassed 93 patients who underwent arthroscopic surgery and observed a notable enhancement in the Karlsson–Peterson score. The score significantly increased from an average preoperative value of 28.3 to a postoperative average of 90.2. Regarding patient satisfaction, 69 out of 73 individuals reported being content with the results, while 4 expressed dissatisfaction [39]. The arthroscopic approach resulted in significantly less early postoperative pain, primarily attributed to its reduced invasiveness and minimal joint capsule dissection compared to the open group [25]. This is consistent with our results. It is widely acknowledged that the open Broström-Gould surgery, a frequently employed procedure for treating CLAI, showed favorable outcomes in the medium term. After a 9-year follow-up, Maffulli et al. [40] reported a significant enhancement in AOFAS scores, increasing them from 51 to 90 points, in patients who underwent open Broström-Gould surgery. Nery et al. [2] utilized the arthroscopic-assisted Broström-Gould procedure to treat CLAI. At a mean follow-up of 9.8 years, 94.7% of patients had postoperative AOFAS scores rated excellent and good. Buerer et al. [41] reported a high satisfaction rate in their study of 41 patients who underwent open modified Broström repair, with an average AOFAS score of 89 at the final follow-up. Molloy et al. [42] investigated the outcomes of open Broström-Gould repair in 21 patients with persistent CLAI. The study showed a significant improvement in AOFAS scores, with the preoperative scores of 53 increasing to 89 after a 25-month follow-up period. Furthermore, several studies have demonstrated that the arthroscopic approach yields favorable treatment outcomes for CLAI [14, 43]. These studies only show the functional scores of open surgery at the final follow-up and do not indicate the postoperative changes over time. Our results showed that arthroscopic repair was superior to open surgical procedure regarding early postoperative functional scores. The superior functional scores in the arthroscopic group can be attributed to the minimal soft tissue dissection. Secondly, the arthroscopic approach minimizes the risk of injuring blood vessels surrounding the ATFL and promotes the repaired ATFL’s vascularization. Conversely, the open group necessitated a more prolonged procedure due to the greater surgical trauma. However, we did not determine whether the differences in functional outcomes at one year exceeded the minimal clinically important difference, or if these differences were clinically relevant. Further research is needed to investigate these aspects.

### Complications

Our findings indicate that there were no significant differences between the two surgical approaches in terms of overall complications and nerve injuries. The literature reports general complication rates ranging from 0 to 35% for arthroscopic lateral ankle ligament repair, while the open technique shows variability with rates between 0 and 29.6% [11]. The complications reported encompass injuries to the superficial peroneal nerve, wound infections, delayed wound healing, deep vein thrombosis, and the occurrence of residual ankle instability. In a comprehensive review comprising six studies, Wang et al. [44] provided an overview of the complication rates associated with arthroscopic repair of lateral ankle instability, which ranged from 0 to 41.9%. Based on previous research findings [33, 45, 46], there is a higher incidence of superficial peroneal nerve complications in arthroscopic surgery. In addition, several studies have reported that the incisions used in open surgery are close to the superficial peroneal nerve, making it vulnerable to injury [28, 47]. However, our study aligns with a previous meta-analysis that reported a similar incidence of nerve injury between the two surgical techniques [33]. The literature has previously documented safety zones in ankle surgeries, showing an average 5.1 cm (3.9–6.4 cm) inter-nervous safe zone between the superficial peroneal nerve and the sural nerve, and an average 4.3 cm (3.7–6.4 cm) inter-tendon safe zone between the peroneus brevis and the peroneus tertius tendon [39]. During arthroscopic surgery, structures in this region were relatively safe from damage caused by portals or suture passages [48]. Our results revealed a higher incidence of nerve injury in the arthroscopic repair group at 4.6%, compared to 2.8% in the open group. However, there was no statistically significant difference between the two surgical approaches. Therefore, nerve injury is not a disadvantage specific to arthroscopic surgery. Using arthroscopic techniques for repair does not result in a higher risk of nerve injury.

### Time efficiency

Additionally, we compared the early postoperative activity time between the two repair techniques and found that arthroscopic repair showed significant advantages over the open group. However, our results indicate no significant difference in the recovery time for regaining complete activity levels between the two fixation approaches. Recently, Hou et al. [25] reported that six months after surgery, the arthroscopic group exhibited a shorter recovery period, higher return rates to athletic activities, and notable improvements in clinical outcomes, muscular strength, and posture control. This may be why some studies show faster recovery from arthroscopic surgery [30, 31]. Our results indicate no significant difference in operation time between the two surgical approaches. However, the result needs to be carefully considered. Experienced surgeons may find that arthroscopic techniques can reduce surgical time due to the absence of skin incision and suturing requirements. Zhou et al. [13] observed that the surgical duration in the first 7 cases was significantly longer compared to the subsequent 24 cases. However, they found that the surgeries in the latter 24 cases could be completed within 45 min. Matsui et al. [30] reported the surgical duration of arthroscopic procedures in their study. They observed that the initial 6 cases had a longer average surgical time of 57.2 min (range 40–95), whereas, in the subsequent 13 patients, the surgical time significantly reduced to an average of 29.6 min (range 22–37). There is a learning curve associated with arthroscopic techniques, and surgeons performing this procedure require adequate training in advance.

Our meta-analysis also had some limitations. First, our study only included two RCTs and did not include more randomized controlled studies of higher methodological quality. This study suggests the need for conducting randomized controlled trials in a multicenter setting to obtain more definitive conclusions. Second, our study did not perform a subgroup analysis on patient weight, which may have influenced the results. Third, our meta-analysis identified the lack of standardization among studies as a significant confounding factor. Fourth, the meta-analysis included studies with varying length follow-up periods, which introduces a potential source of heterogeneity. These factors may impact the reliability and stability of the conclusions drawn from our meta-analysis. Fifth, there may be technical heterogeneity among the included studies, which could impact our results.

## Conclusion

Our findings support that arthroscopic repair yields comparable outcomes to open surgery. Consequently, we advocate for adopting arthroscopic repair as a preferred alternative to the conventional open Broström-Gould procedure for treating chronic lateral ankle instability.

## Data Availability

All data generated or analysed during this study are included in this article.

## Abbreviations

CLAI: Chronic lateral ankle instability
ATFL: Anterior talofibular ligament
RCT: Randomised controlled trials
NOS: Newcastle–Ottawa scale
OR: Odds ratios
CI: Confidence interval
WMD: Weighted mean difference
